# Upconversion Modulation through Pulsed Laser Excitation for Anti-counterfeiting

**DOI:** 10.1038/s41598-017-01611-9

**Published:** 2017-05-02

**Authors:** Yingdong Han, Hongyu Li, Yangbo Wang, Yue Pan, Ling Huang, Feng Song, Wei Huang

**Affiliations:** 1 School of Physics & The Key Laboratory of Weak Light Nonlinear Photonics, Ministry of Education, Nankai University, Tianjin, 300071 China; 2 Key Laboratory of Flexible Electronics (KLOFE) & Institute of Advanced Materials (IAM), Jiangsu National Synergetic Innovation Center for Advanced Materials (SICAM), Nanjing Tech University (NanjingTech), 30 South Puzhu Road, Nanjing, 211816 P.R. China; 3 School of Chemistry and Chemical Engineering, Inner Mongolia University, 235 West Daxue Road, Hohhot, 010021 P.R. China

## Abstract

Lanthanide-doped upconversion nanomaterials are emerging as promising candidates in optoelectronics, volumetric display, anti-counterfeiting as well as biological imaging and therapy. Typical modulations of upconversion through chemical methods, such as controlling phase, composition, morphology and size enable us to rationally manipulate emission profiles and lifetimes of lanthanide ions by using continuous-wave laser excitation. Here we demonstrate that under pulsed laser excitation the emission color of NaYF_4_:Er/Tm (2/0.5%)@NaYF_4_ core-shell nanoparticles has an obvious transformation from green to red colors. Moreover, both pulse duration and repetition frequency are responsible for manipulating the upconversion emission color. The mechanism of the phenomena may be that the pulsed laser sequence triggers the emission levels to non-steady upconversion states first, and then cuts off the unfinished population process within the pulse duration. This pump source dependent and resultant tunable fluorescence emission enables NaYF_4_:Er/Tm (2/0.5%)@NaYF_4_ nanoparticles as a promising fluorophore in the transparent anti-fake printing.

## Introduction

Lanthanide ions (Ln^3+^)-based upconversion nanoparticles (UCNPs), which are able to harvest two or multiple low-energy photons and convert the energy to a high-frequency photon, are becoming powerful functionalized materials in many applications including volumetric displaying, optical data storage, photovoltaics and bioimaging^1–3^. Unlike upconversion emissions achieved by simultaneous two-photon absorption and second harmonic generation, Ln^3+^-based upconversion involves real metastable intermediate states, which in turn significantly improve the conversion efficiency while merely demanding moderate pumping laser power density (1–10^3^ w/cm^2^)^4^. Moreover, the fluorescence of UCNPs is resistant with detrimental photobleaching and scintillation. Among all of the reported upconversion host materials, hexagonal-phase NaYF_4_ (β-NaYF_4_) possesses the highest upconversion quantum efficiency due to its low phonon energy (<450 cm^−1^)^5^, which effectively diminishes the non-radiative transition rates. The pursuing of rationally controlling unique emission properties (wavelength, intensity and lifetime) of UCNPs, which in turn gives rise to instructions of UCNPs’ application in bioimaging, three-dimensional display, optical information storage, telecommunication and anti-counterfeiting, drives us to investigate strategies of affecting the upconversion process of lanthanides ions. Conventional approaches control the emission profile by adjusting phase^6^, concentration^7^, size^8^, doping category^9^ and structure^10^ to affect the upconversion dynamic process, and then blend different types of materials with three primary colors, red-green-blue (RGB), with proper portion to get the prescribed color. However, it is always challenging to obtain a tunable emission color in one fixed material. Liu’s group reported that the emission color of NaYF_4_:Nd/Yb@NaYF_4_:Yb/Tm@NaYF_4_@NaYF_4_:Yb/Ho/Ce@NaYF_4_ core-multishell nanoparticles could change from green to red by prolonging pump pulse duration of a single-wavelength laser diode^11^. Then researchers found that the fluorescence green-to-red ratio (GRR) depends on the pulsed excitation and the crystal with a higher phonon energy is more sensitive to the pulsed duration^12^. Beyond the tunable emission color or peak ratio resulted from controlling pulse duration, the pulsed laser excitation brings about higher quantum yield than continuous-wave (CW) laser with equivalent average power density, enabling deep tissue optical bioimaging^13, 14^. However, new type of color-shifting UCNPs is still limited and comprehensive understanding of the upconversion modulation discipline by pulsed laser especially for its repetition frequency has not realized until now.

Herein we designed an active core-inert shell structured nanocrystal with the composition of NaYF_4_:Er/Tm (2/0.5%)@NaYF_4_ to obtain an insight into the modulation mechanism on upconversion emission by pulse duration and repetition frequency, respectively. Selecting the Er/Tm ion pair was based on its pure red emission in hexagonal phase NaYF_4_ nanoparticles under CW laser excitation. Actually, the GRR of nanoparticles with prominent green emission under CW laser excitation, such as β-NaYF_4_:Yb/Er (20/2%) nanocrystals, could also be changed under pulsed laser excitation. Nevertheless, the emission color is always located in the green region. In our design, under short pulse duration and low repetition frequency 980 nm near infrared (NIR) laser excitation, the as-synthesized NaYF_4_:Er/Tm (2/0.5%)@NaYF_4_ nanoparticles exhibited green emission, which is different from its typical red emission under CW 980 nm laser excitation. We found that the GRR evidently changed with pulse duration and repetition frequency, respectively. This significantly improves the anti-fake quality when using NaYF_4_:2Er/0.5Tm@NaYF_4_ as printing ink. Our proposed mechanism considered that the upconversion temporal population and depopulation proceeding which are influenced by pulse duration and repetition frequency give rise to combined impacts on the ultimate luminescence profile.

## Results and Discussion

**Figure 1:**
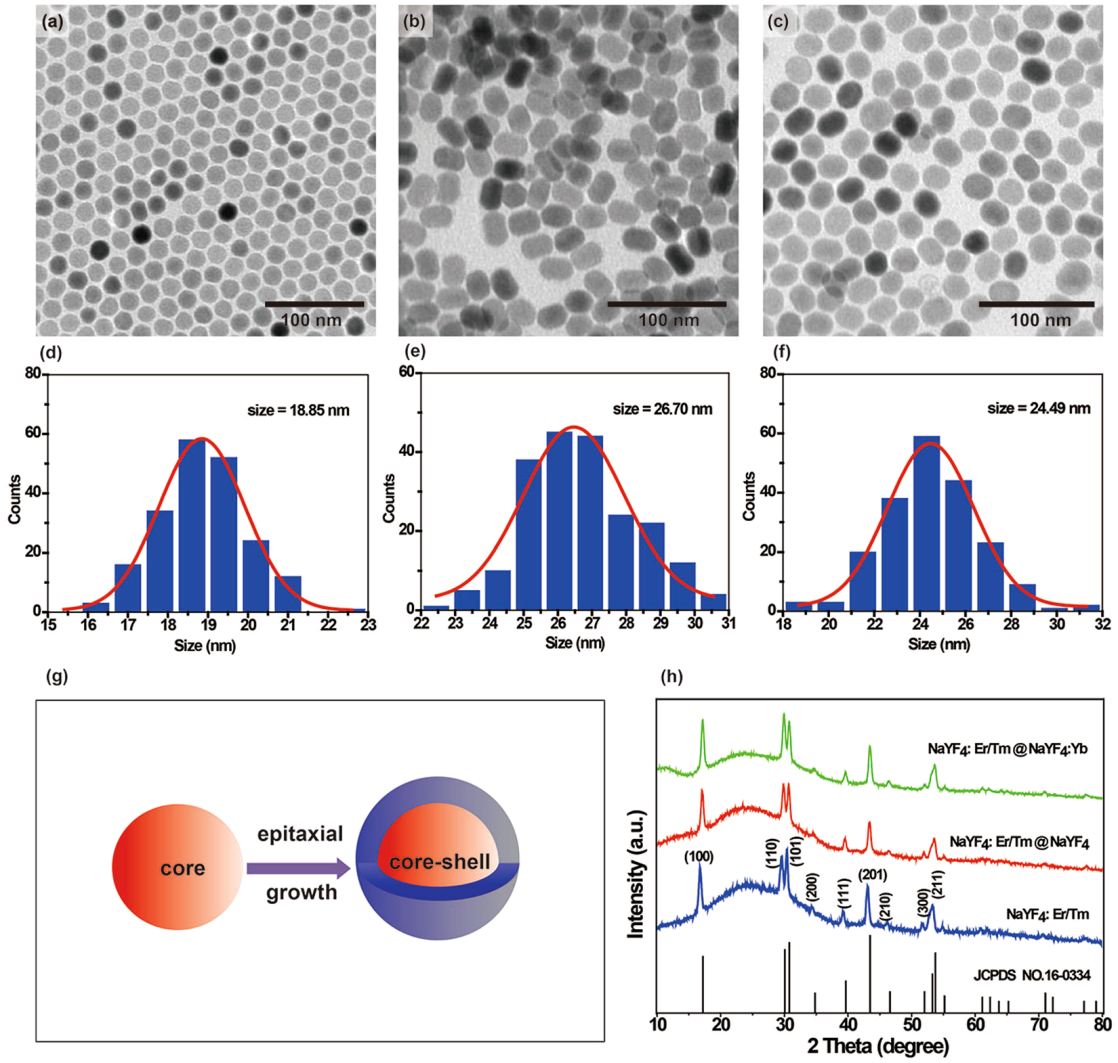
Composition and morphology characterization of the as-prepared nanoparticles. (**a–c**) TEM images of the as-synthesized nanoparticles: (**a**) NaYF_4_:Er/Tm (2/0.5%), (**b**) NaYF_4_:Er/Tm(2/0.5%)@NaYF_4_ and (**c**) NaYF_4_:Er/Tm(2/0.5%)@NaYF_4_:Yb (20%); (**d–f**) The size distribution of the corresponding nanoparticles: (**d**) NaYF_4_:Er/Tm (2/0.5%), (**e**) NaYF_4_:Er/Tm(2/0.5%)@NaYF_4_ and (**f**) NaYF_4_:Er/Tm(2/0.5%)@NaYF_4_:Yb (20%); (**g**) Schematic procedure for the synthesis of hexagonal NaYF_4_-based core-shell nanoparticles; (**h**) XRD patterns of the NaYF_4_:Er/Tm(2/0.5%), NaYF_4_:Er/Tm(2/0.5%)@NaYF_4_ and NaYF_4_:Er/Tm(2/0.5%)@NaYF_4_:Yb (20%) nanoparticles as indexed in accordance with hexagonal-phase NaYF_4_ crystal structure (Joint Committee on Powder Diffraction Standards file No. 16-0334).

For illustration, to obtain high emission intensity as well as maintain red emission color under CW laser excitation, NaYF_4_:Er/Tm (2/0.5%)nanoparticle and its core-shell structures, i.e., NaYF_4_:Er/Tm (2/0.5%)@NaYF_4_ and NaYF_4_:Er/Tm (2/0.5%)@NaYF_4_:Yb (20%) nanoparticles, were all synthesized through thermal co-precipitation method. According to transmission electron microscopy (TEM) images (Fig. 1(a–c) and Figure S1), all of the as-synthesized nanoparticles were monodisperse in cyclohexane solution with uniform diameters. As is shown in the size distribution graph in Fig. 1(d), the average size of NaYF_4_:Er/Tm nanocrystal is 18.85 nm. After subsequent shell-coating procedures, the sizes of NaYF_4_:Er/Tm@NaYF_4_ and NaYF_4_:Er/Tm@NaYF_4_:Yb (Fig. 1(e) and (f)) increased to 26.70 nm and 24.49 nm, respectively, suggesting successful epitaxial growth of shell onto the core (Fig. 1(g)). X-ray diffraction (XRD) data, shown in Fig. 1(h) and Figure S2, clearly indicates that these samples are all indexed to hexagonal phase NaYF_4_ nanocrystals.

**Figure 2:**
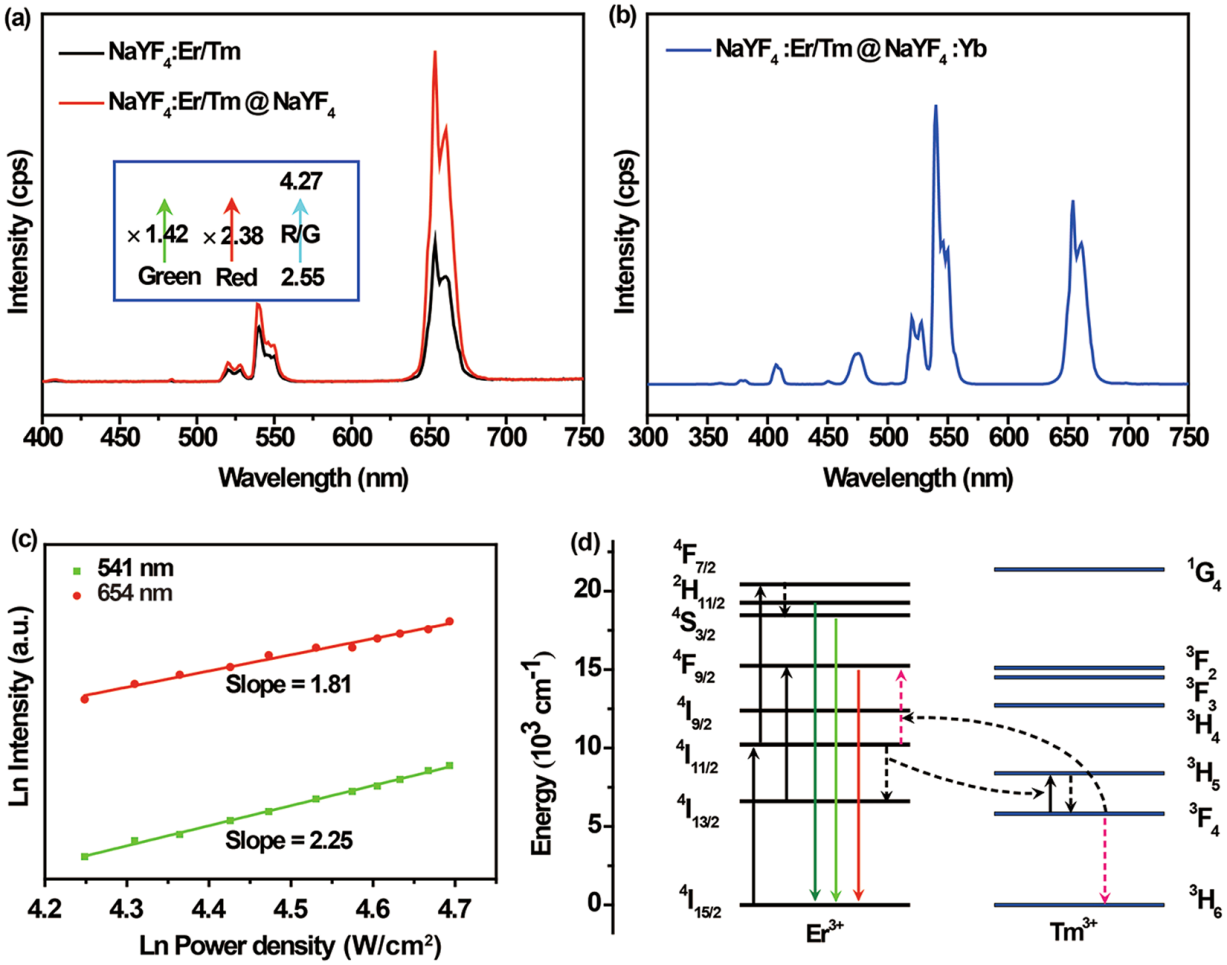
Photoluminescence features of the upconversion nanoparticles. (**a–b**) The emission spectra of nanoparticles under CW 980 nm diode laser excitation: (**a**) NaYF_4_:Er/Tm, NaYF_4_:Er/Tm@NaYF_4_ and (**b**) NaYF_4_:Er/Tm@NaYF_4_:Yb; (**c**) Excitation power density dependence at 541 nm and 654 nm of NaYF_4_:Er/Tm@NaYF_4_ nanocrystals under CW 980 nm laser excitation; (**d**) Proposed energy transfer upconversion mechanism of NaYF_4_:Er/Tm@NaYF_4_ nanoparticles.

The typical upconversion spectra of the as-prepared three kinds of nanoparticles with equivalent mass concentration (1 mg/mL) were measured upon continuous 980 nm laser excitation (42 W/cm^2^). As is shown in Fig. 2(a), the upconversion spectra of NaYF_4_:Er/Tm (2/0.5%) and NaYF_4_:Er/Tm (2/0.5%)@NaYF_4_ upon CW 980 nm laser excitation gave rise to three dominant peaks at 520 nm, 541 nm and 654 nm, which are attributed to optical transitions of Er^3+^: ^2^H_11/2_ → 4I_15/2_, ^4^S_3/2_ → 4I_15/2_ and ^4^F_9/2_ → 4I_15/2_, respectively. The doping Tm^3+^ ions here interfere with Er^3+^ ions’ upconversion process by introducing a recycling energy transfer pathway, which contains transitions of ^4^I_11/2_(Er^3+^) + ^3^F_4_(Tm^3+^) → ^4^I_13/2_(Er^3+^) + ^3^H_5_(Tm^3+^) and ^3^F_4_(Tm^3+^) + ^4^I_11/2_(Er^3+^) → ^4^F_9/2_(Er^3+^) + ^3^H_6_ (Tm^3+^), to facilitate red emission^15, 16^ (Fig. 2(d)). The overall emission intensity of NaYF_4_:Er/Tm nanocrystals was remarkably enhanced after NaYF_4_ shell coating owing to elimination of quenchers on the surface of NaYF_4_:Er/Tm nanoparticles^10, 17^. Note that the enhancement factor for red emission is more prominent than that of the green emission, further elevating the red to green ratio. This effect implies that more activators participate in the recycling energy transfer procedure rather than are captured by surface quenchers after constructing the core-shell structure. Moreover, except for adapting inert shell (NaYF_4_ shell layer) to improve photoluminescence efficiency, we also tried to coat active shell (NaYF_4_:Yb shell layer) onto the NaYF_4_:Er/Tm core. However, the Yb^3+^ ions, which possess the largest cross-section at 980 nm among all lanthanide ions, absorbed pump photons first, and then transferred the energy to Er^3+^ and Tm^3+^ ions individually. As a result, the upconversion route and the emission spectra (Fig. 2(b)) changed, thus violating our pre-demand of red emission color under CW laser excitation. Herein NaYF_4_:Er/Tm@NaYF_4_ is the most suitable sample in our design. The slopes of ln–ln plots of emission intensity at 541 nm and 654 nm versus 980 nm excitation power density clearly indicate that the upconversion populations of ^4^S_3/2_ and ^4^F_9/2_ energy levels are both realized through two-photon upconversion process (Fig. 2(c)).

**Figure 3:**
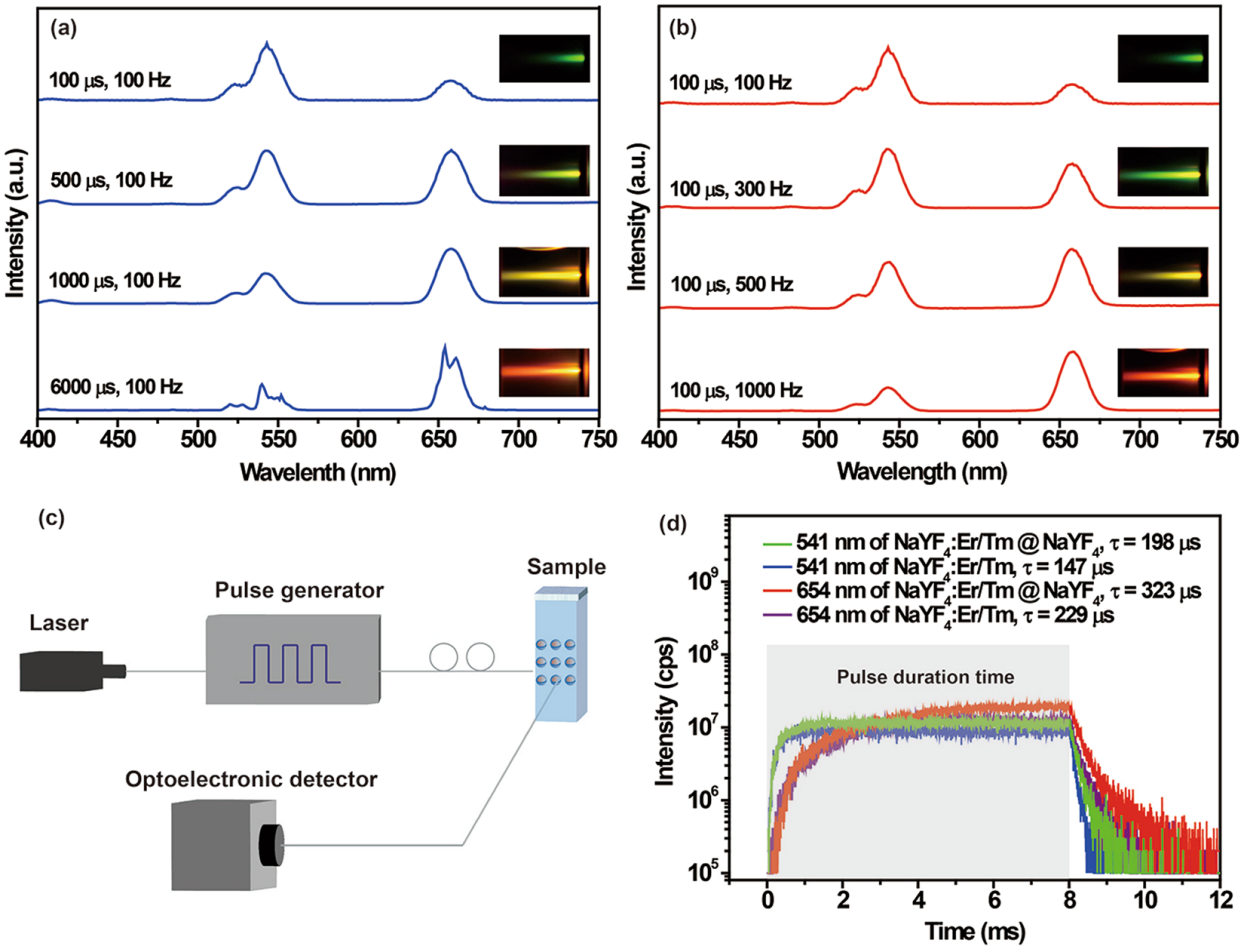
Temporal control of upconversion luminescence. (**a–b**) Photoluminescence spectra of NaYF_4_:Er/Tm@NaYF_4_ nanocrystals under pulse 980 nm diode laser excitation: (**a**) x µs, 100 Hz (x = 100, 500, 1000, 6000) and (**b**) 100 µs, y Hz (y = 100, 300, 500, 1000); (**c**) Experimental setup for upconversion spectra detection under pulsed 980 nm laser excitation and lifetime measurement; (**d**) Time-dependent evolutions of emission intensity at 541 nm and 654 nm of NaYF_4_:Er/Tm and NaYF_4_:Er/Tm@NaYF_4_ nanocrystals showing the rising time and decay curve.

**Figure 4:**
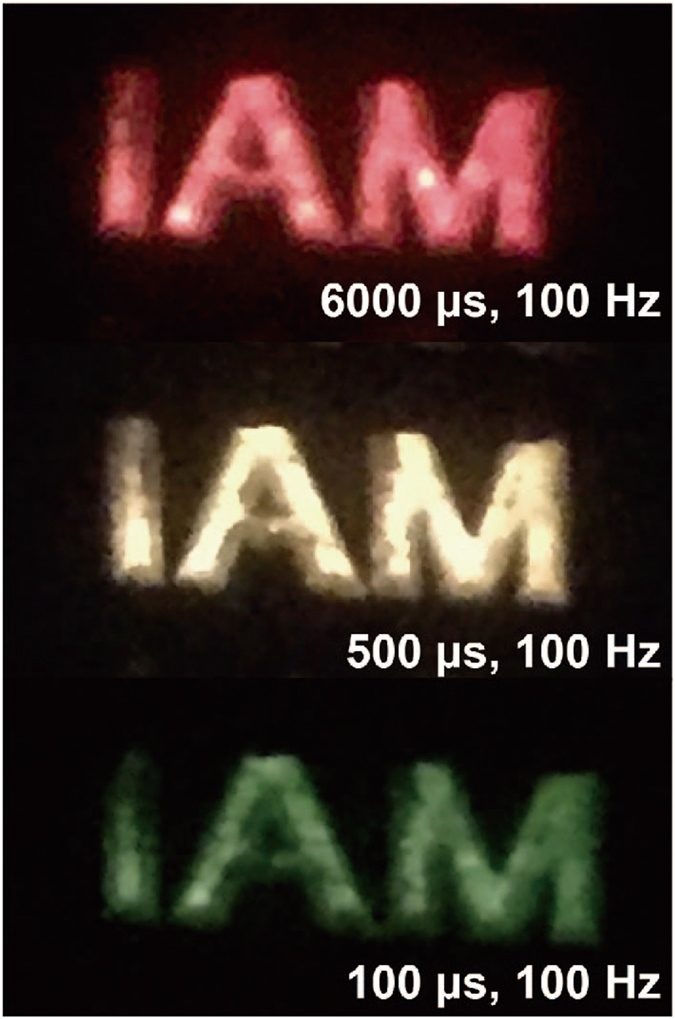
The photograph of tunable emission color of NaYF_4_:Er/Tm@NaYF_4_ nanoparticles under pulsed laser excitation for anti-counterfeiting. The size of one character is 3 mm × 3 mm.

As for the upconversion spectra measurement of NaYF_4_:Er/Tm@NaYF_4_ nanoparticles under pulsed laser excitation, firstly the pulse duration time was set within the range from 100 µs to 6 ms, while the repetition frequency was fixed at 100 Hz. Figure 3(c) is the schematic diagram illustrating the experimental setup for upconversion spectra measurement. The pulse generator was used to shape 980 nm NIR laser with controllable pulse duration time and repetition frequency and afterwards send the modulated laser to upconversion nanocrystals, which generate spectrum information captured by the optoelectronic detector. It is clearly depicted in Fig. 3(a) that by gradually prolonging the pulse width the emission color was adjusted from green, yellow to red. We further checked the emitting color dependence on frequency under permanent pulse duration. It was found that the emission color was also tuned from green to red with the frequency increasing meanwhile the pulse duration was set as 100 µs (Fig. 3(b)). When the pulse duration was longer, the frequency adjusting effect on emission color displayed the same tendency but in a narrow scope. These results indicated that both pulse width and frequency were able to manipulate the emission color of NaYF_4_:Er/Tm@NaYF_4_ nanoparticles and under excitation of short pulse duration and low frequency laser the green emission was ‘extracted’ from upconversion process while the red emission was suppressed. It was noted that when pulse duration was longer or the repetition frequency was higher, the emission intensity became stronger as a result of enhanced pump power density. Due to the special multicolor emission features in homogeneous materials, the core–shell nanoparticles were designed as transparent fluorescence ink for improving anti-counterfeiting level. A series of character patterns were firstly embed into a PMMA plate through laser ablation. The cyclohexane solution with NaYF_4_:Er/Tm@NaYF_4_ dispersion was injected into a part of character patterns. In ambient lighting, all the character patterns seem identical. However, under 980 nm laser excitation (CW or long pulse), only the labelled patterns (‘IAM’) exhibited red emission (Fig. 4). Significantly, as gradually adjusting the pulsed laser conditions, the ‘IAM’ patterns changed to yellow and green color. This unique property greatly enhanced the anti-counterfeiting level.

To understand color modulation mechanism through pulsed diode laser excitation, the time-dependent evolutions of emission intensity at 541 nm and 654 nm of as-prepared NaYF_4_:Er/Tm and NaYF_4_:Er/Tm@NaYF_4_ nanoparticles were investigated under 980 nm laser excitation with 8 ms duration time and 20 Hz repetition rate. As is indicated in Fig. 3(d), in the pulse duration time the 541 nm and 654 nm emission of the as-synthesized nanoparticles rise to their steady states within several milliseconds, as the populations of the emission energy levels are involved with sequential optical transitions. We found that without Tm ions the upconversion sample would give rise to green emission color (Figure S3). Moreover, after Tm ions doping the rising evolutions of green and red emission were more evidently separated (Figure S4), which was probably ascribed to the fact that two energy transfer processes participate in ^4^F_9/2_ population process. The separation of rising section makes it possible to timely activate the emission levels using modulated pulsed laser. As a consequence, under short pulse width excitation and a relatively low frequency the green emission intensity is stronger than the red intensity. When the pulse duration is prolonged while fixing the repetition frequency at 100 Hz, the red emission follows a gradual approach to its steady equilibrium state, yielding enhanced emission intensity. Therefore, the emission color changed from green to yellow and finally to red with pulse duration increasing. The lifetime of upconversion nanocrystals can be calculated based on the decay curve. Typically, the lifetime curve is fitted with the equation:

$${\rm{I}}({\rm{t}})={{\rm{I}}}_{0}+{\rm{e}}(-({\rm{t}}-{{\rm{t}}}_{0})/{\rm{\tau }})$$

where I_0_ is emission intensity at the initial decay time t_0_ and τ is the calculated lifetime of the emission energy level. Therefore, the fitted lifetimes of red emission and green emission in NaYF_4_:Er/Tm@NaYF_4_ are 323 µs and 198 us, respectively. It is worth noting that, as displayed in Fig. 3, lifetimes of 541 nm and 654 nm emissions are prolonged after shell coating, verifying the enhanced luminescence intensity in NaYF_4_:Er/Tm@NaYF_4_ compared to NaYF_4_:Er/Tm.

**Figure 5:**
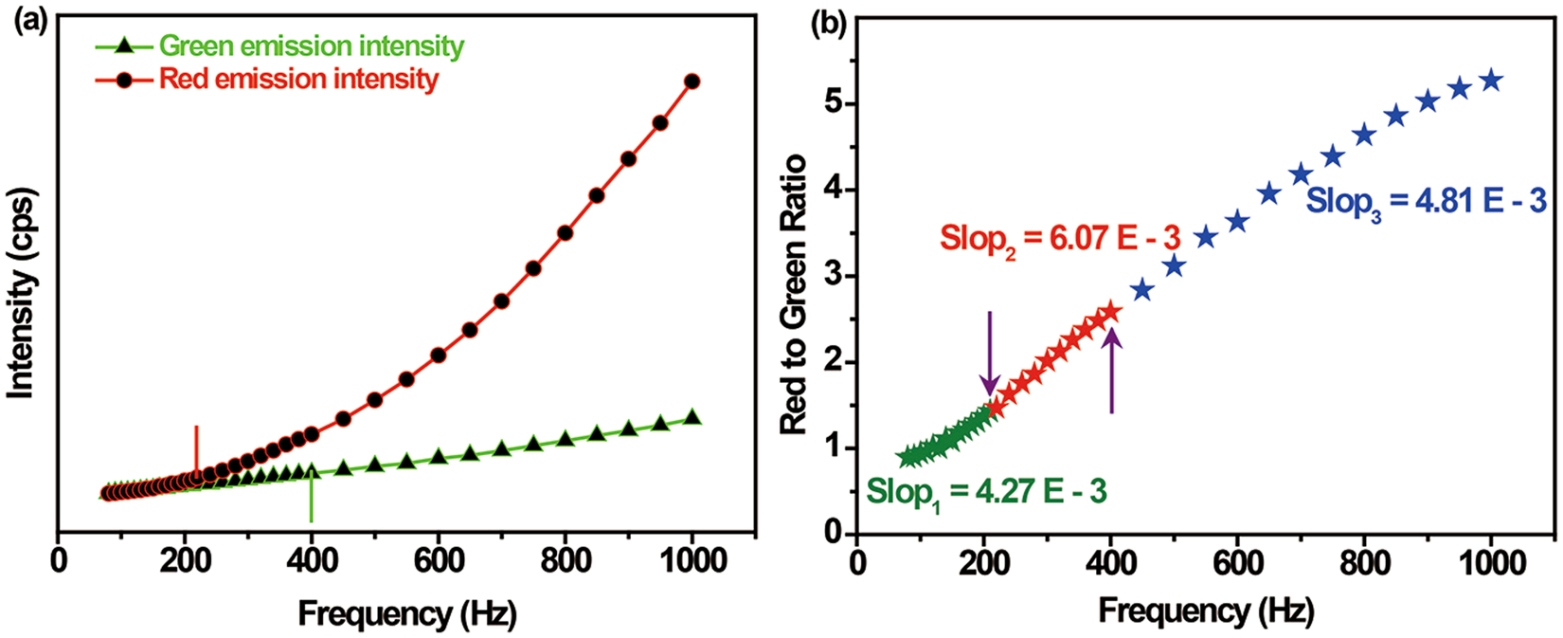
The effect of repetition frequency on upconversion emission. (**a**) The green emission at 541 nm and red emission at 654 nm intensity; (**b**) Red-to-green ratio dependence on repetition frequency when the pulse duration is fixed as 500 µs.

We next investigated how the repetition frequency influences GRR under fixed pulse duration. It is convinced that the pulse duration time is not the only reason for tuning the GRR, otherwise the GRR will be permanent with frequency changing. As is indicated by the luminescence decay curves in Fig. 3(d), the red emission and green emission take 4 ms and 2 ms for their complete deactivation to initial states after the excitation source is shut down. The depopulation time is independent on pulse width because it is the nature character of the energy level. For illustrating, our frequency-dependent emission experiments, shown in Fig. 5(a), indicate that the red emission intensity is more sensitive to frequency than the green under pulsed 980 nm NIR laser excitation. The frequency effect on red or green emission intensity can be divided into two sections, which are separated by the time points that the intervals between two pulses are just equal to their complete deactivation time. When the pulse width is 500 µs, at which point the green emission level is close to its maximum population whereas the red emission is at its initial population state (Fig. 3(d)), the critical frequencies for red and green emission are therefore 220 Hz (~1/(4 ms + 500 µs)) and 400 Hz (1/(2 ms + 500 µs)), respectively. It is found that separated by their critical frequencies the red emission and green emission intensities grow with different rising gradients. The regular pattern of intensity dependence on frequency is further verified by red to green ratio changing with frequency (shown in Fig. 5(b)). It can be concluded from Fig. 5(b) that the slope of red to green ratio are 4.27 × 10^−3^, 6.07 × 10^−3^ and 4.81 × 10^−3^ when the frequency located in the scale of <220 Hz, 220 Hz < frequency < 400 Hz and frequency > 400 Hz, respectively. Obviously, the slope of red to green ratio presented a slight decrease after a slight increase. This phenomenon can be interpreted by the fact that with frequency increasing the subsequent pulse first reaches the complete deactivation point of red emission level and afterwards the red emission intensity begins to increase faster whereas the green emission intensity evolution is still at its slow increment scale (<400 Hz). When the repetition frequency exceeds 400 Hz, the green emission intensity starts to rise faster leading to depression of red to green ratio slope.

## Conclusion

Traditional photon upconversion in lanthanide ions doped materials are usually realized through CW laser excitation, thus the emission color of one fixed type nanoparticle is typically monotonous merely with minor change. We here demonstrated that an identical sample, NaYF_4_:Er/Tm (2/0.5%)@NaYF_4_ core-shell structured nanocrystal, could display green, yellow and red emission colors, which depends on elaborately manipulating the pulse duration or repetition frequency of the pumping laser. The microsecond-scale short pulse duration of pumping laser triggered population separation of green and red emission energy levels before reaching steady states due to their differentiable rising time. And the pulse repetition frequency affects emission evolution with different tendency depending on whether the time between two pulses is longer than its absolute decay time or not. Therefore, we attributed the upconversion emission modulation to a synthetical effect derived from rising and decay nature of emission levels, which are influenced by the pulsed laser. Our study showed that the NaYF_4_:Er/Tm@NaYF_4_ core-shell upconversion nanocrystal could be a promising fluorescent substance for invisible and color-tunable anti-counterfeiting ink.

## Methods

### Experiment materials

Yttrium(III) acetate hydrate (99.9%), erbium(III) acetate hydrate (99.9%), thulium acetate hydrate (99.9%), ytterbium(III) acetate hydrate (99.9%), oleic acid (90%) and 1-octadecene (90%) were purchased from Alfa Aesar. Sodium hydroxide (NaOH, >96%), ammonium fluoride (NH_4_F, >96%), cyclohexane(99.5%), ethanol(99.7%) and methanol(99.5%) were purchased from Shanghai Lingfeng chemical reagent Co. Ltd. and used as received unless otherwise noted.

### Synthesis method

The synthesis procedure is similar to that utilized in previous work^18^ only with minor modifications.

#### Synthesis of NaYF_4_: Er/Tm (2/0.5 mol%) Nanoparticles

In a typical experiment, 2 mL of water solution containing Y(CH_3_CO_2_)_3_ (0.39 mmol), Er(CH_3_CO_2_)_3_ (0.008 mmol) and Tm(CH_3_CO_2_)_3_ (0.002 mmol) were pipetted into a 100-mL flask, then 3 mL oleic acid and 7 mL 1-octadecene were added into the flask. The resulting mixed liquid was heated at 150 °C for 1 h with stirring and then cooled down to room temperature. Thereafter, 2 mL methanol solution containing NaOH (0.5 mmol/mL) and 2 mL methanol solution with NH_4_F (0.4 mmol/mL) were added into the flask and stirred at 40 °C for 30 min, after which time the mixture was heated to 90 °C to remove the methanol. After eliminating bubbles in the liquid, the solution was heated to 290 °C and kept at this temperature for 1.5 h in an argon air atmosphere. Finally, the mixture was cooled down slowly to room temperature. The resulting liquid was washed with ethanol and cyclohexane for 3 times, collected by centrifugation, and re-dispersed in 5 mL of cyclohexane.

#### Synthesis of NaYF_4_:Er/Tm (2/0.5 mol%)@NaYF_4_:Yb (x mol%) (x = 0, 20) Core-Shell Nanoparticles

Firstly, 1 mL of water solution containing Y(CH_3_CO_2_)_3_ and Yb(CH_3_CO_2_)_3_ with a total lanthanide amount of 0.2 mmol, were pipette into a 100-mL flask. The precise amounts of Y(CH_3_CO_2_)_3_ and Yb(CH_3_CO_2_)_3_ were calculated depending on their mole ratio in the shell layer. And then we added 3 mL oleic acid and 7 mL 1-octadecene into the flask. The resulting mixture was heated at 150 °C for 1 h with stirring and then cooled down to room temperature. Subsequently, we added as-synthesized 2.5 mL cyclohexane solution containing NaYF_4_:Er/Tm nanoparticles and methanol solution of NaOH (1 mL, 0.5 mmol/L) and NH_4_F (2 mL, 0.4 mmol/L) into the flask. The following treatment to the mixture is the same as the method for synthesizing NaYF_4_:Er/Tm nanoparticles except for dispersing the product in 2.5 mL cyclohexane finally.

#### Synthesis of NaYF_4_:Er (2 mol%) and NaYF_4_:Er (2 mol%)@NaYF_4_ Nanoparticles

To understand the upconversion process, these two types of nanoparticles were prepared using the same method displayed above.

### Data availability statement

The datasets generated during and/or analysed during the current study are available from the corresponding author on reasonable request.

### Characterization

XRD patterns were obtained by a Rigaku D/max 2550 X-ray diffractometer using CuKa radiation (λ = 0.154 nm). TEM images were performed on a Hitachi 7700 transmission electron microscope operating at 200 kV. UC luminescence spectra and decay curves were recorded on a Fluorolog^®^-3 Spectrofluorometer by Horiba using an external (continuous or pulsed) 980 nm diode laser as the excitation source. All measurements were taken under room temperature.

### Anti-counterfeiting Preparation

To show the anti-counterfeiting ability of NaYF_4_:Er/Tm@NaYF_4_ nanocrystals, the cyclohexane solution containing NaYF_4_:Er/Tm@NaYF_4_ was used as ink to be injected into the character marks in the polymethyl methacrylate (PMMA) plate. The security information was read out by a camera when using pulsed 980 nm laser to excite the labeled characters.

## Electronic supplementary material

supplementary information
