# Supplementary material for: Upconversion Modulation through Pulsed Laser Excitation for Anti-counterfeiting
Source: Sci Rep. 2017 May 2;7:1320. doi: 10.1038/s41598-017-01611-9 (PMC5430986; doi:10.1038/s41598-017-01611-9)
Supplement: Supplementary file 1 — supplementary information [file 41598_2017_1611_MOESM1_ESM.doc]

**Supplementary Information**

**Upconversion Modulation through Pulsed Laser Excitation for Anti-counterfeiting**

Yingdong Han, Hongyu Li, Yangbo Wang, Yue Pan, Ling Huang, Feng Song, Wei Huang


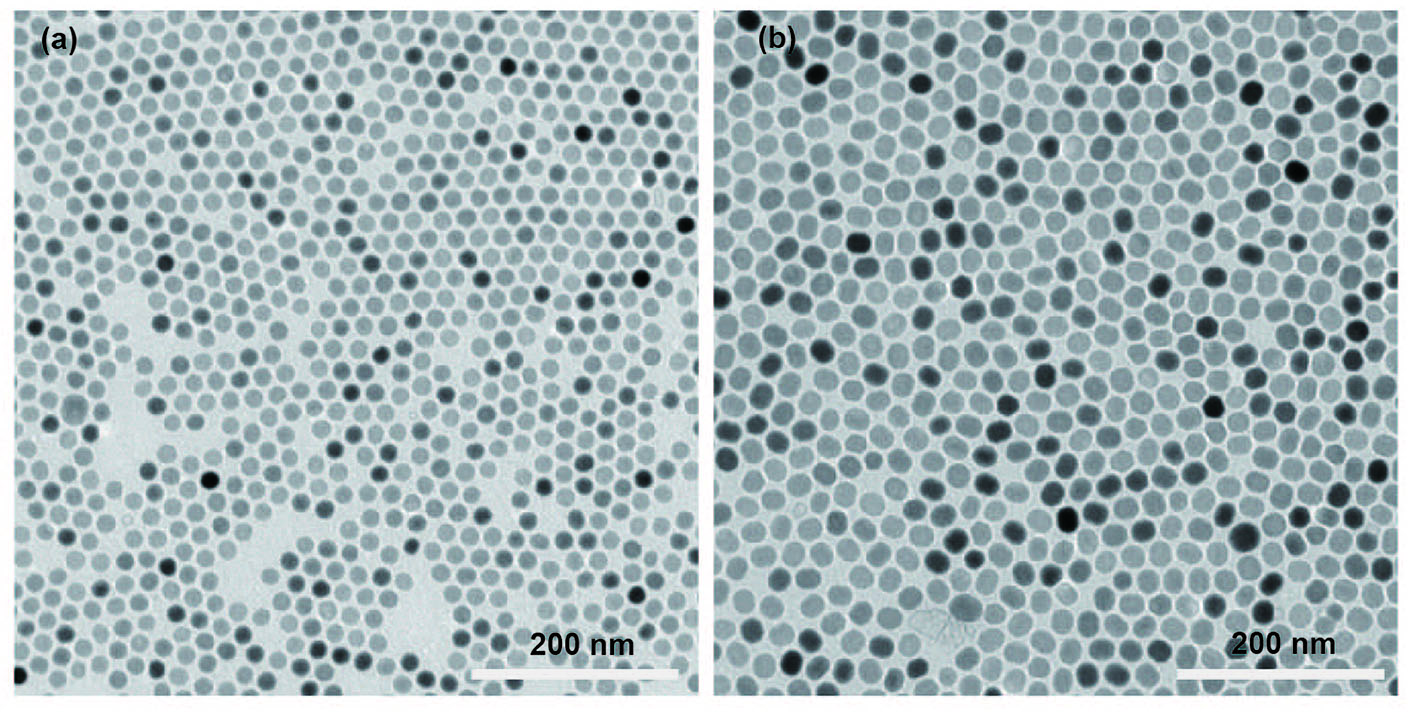


Figure S1 TEM images of the as-synthesized samples: (a) NaYF_4_:Er (2 mol%) and (b) NaYF_4_:Er (2 mol%)@NaYF_4_ nanoparticles.


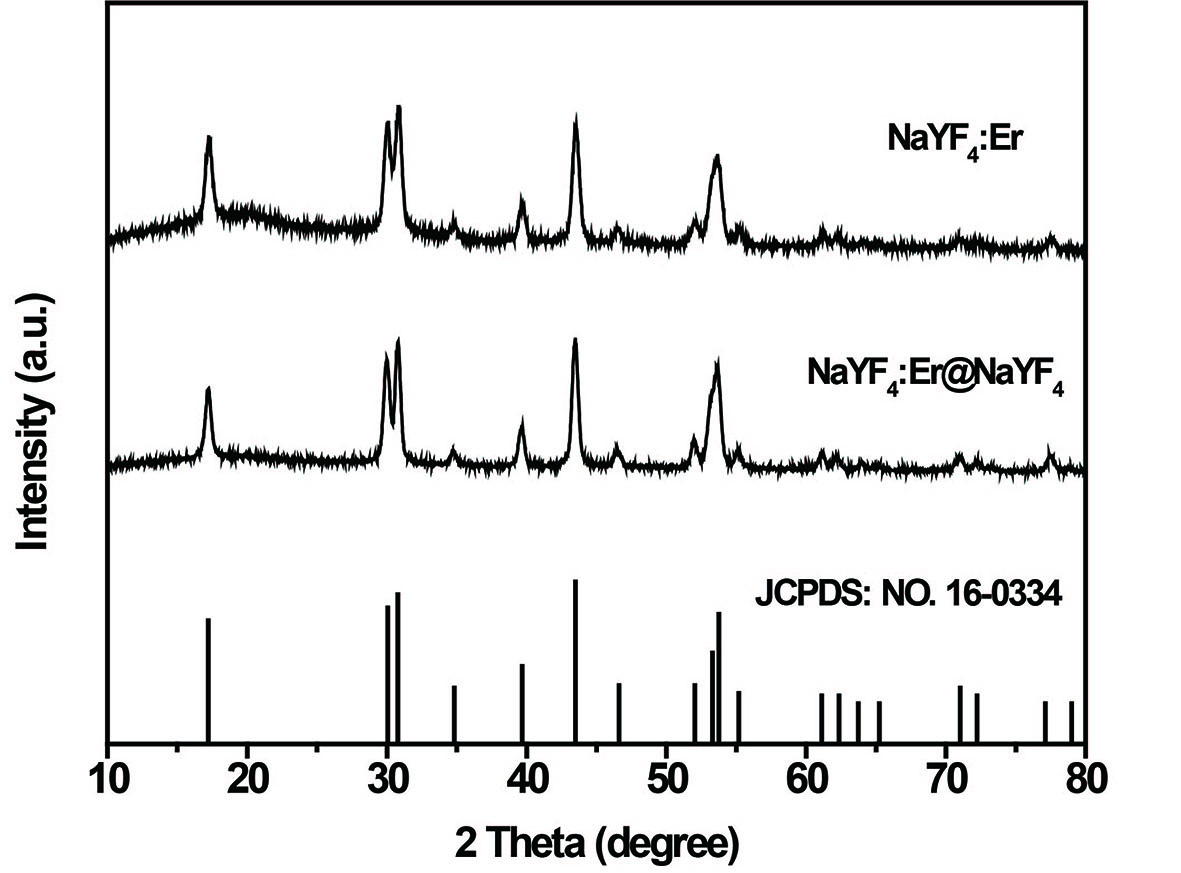


Figure S2 XRD characterization of the as-prepared NaYF_4_:Er (2 mol%) and NaYF_4_:Er (2 mol%)@NaYF_4_ core-shell upconversion nanocrystals indicating they are indexed to hexagonal phase NaYF_4_ nanocrystals (JCPDS: NO. 16-0334).


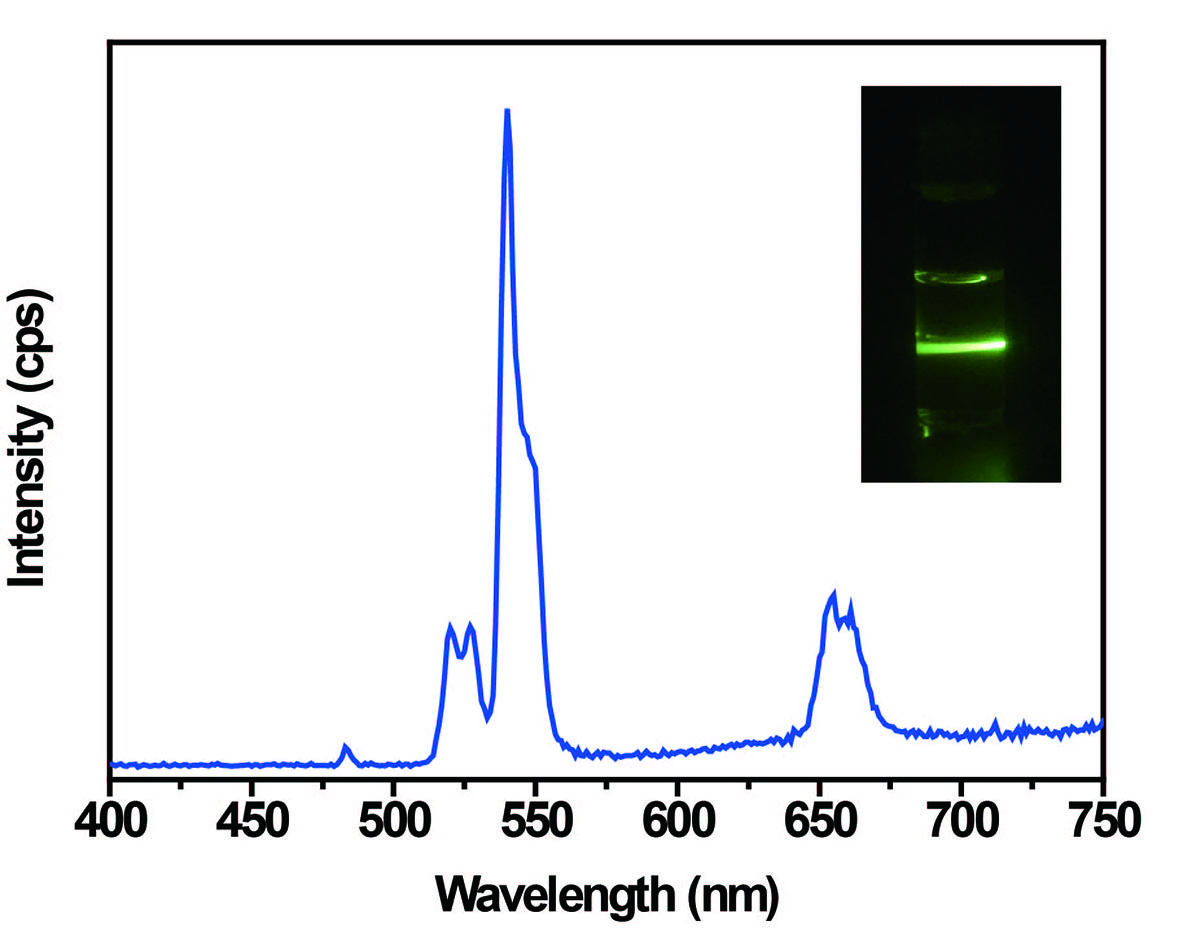


Figure S3 Photoluminescence spectra of NaYF_4_:Er (2 mol%)@NaYF_4_ nanoparticles under 980 nm laser excitation. The inset is the corresponding photograph.


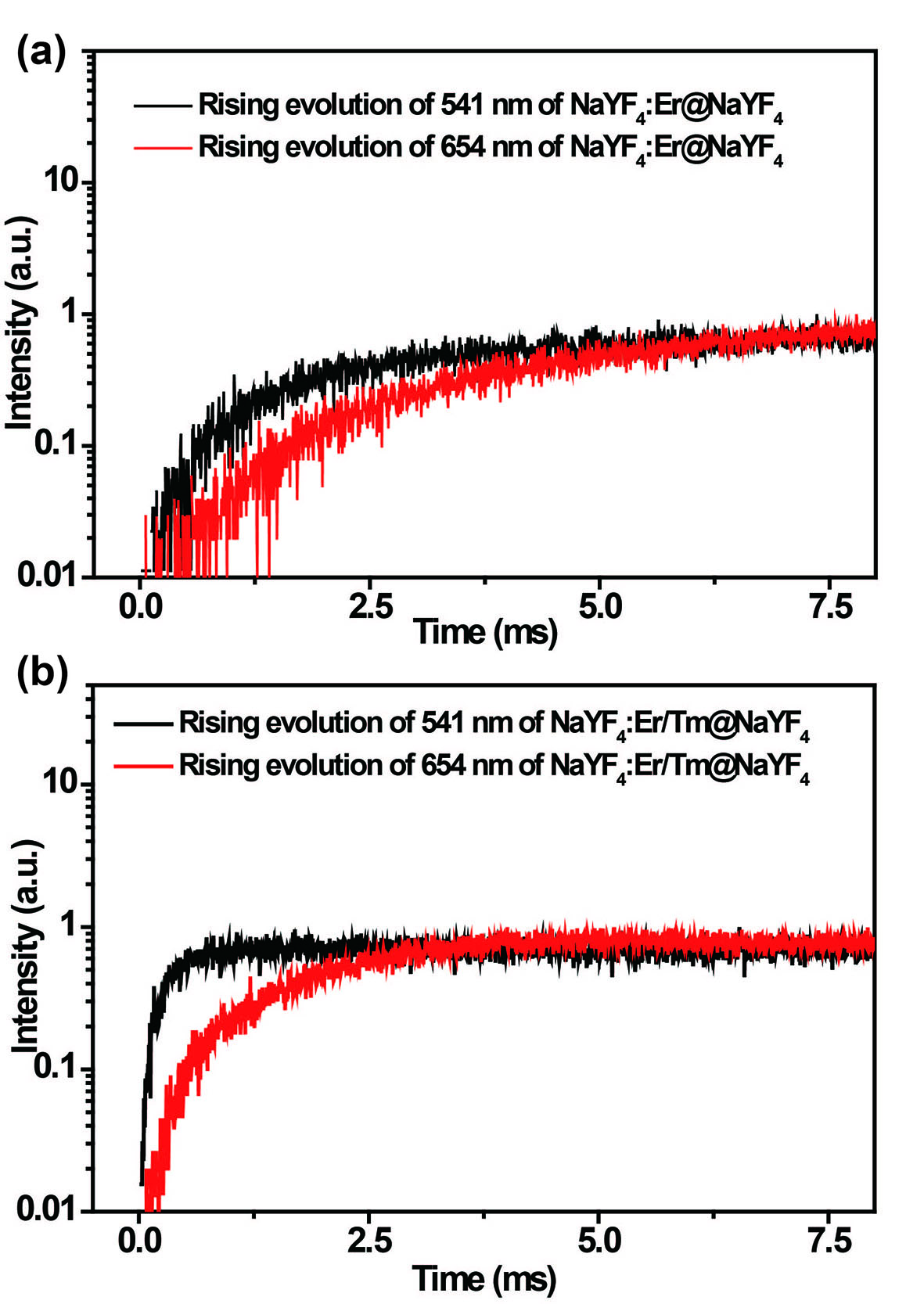


Figure S4 Rising evolutions of green and red emission of as-synthesized nanoparticles without (a) and with (b) Tm ions.
